# Dentists’ awareness of data security and ethical issues during the transition to artificial intelligence-driven clinical practice

**DOI:** 10.2340/aos.v85.45419

**Published:** 2026-02-04

**Authors:** Furkan Ozbey, Busra Nur Gokkurt Yilmaz, Birkan Eyup Yilmaz

**Affiliations:** a Department of Dentomaxillofacial Radiology, Faculty of Dentistry, Afyonkarahisar Health Sciences University, Afyonkarahisar, Türkiye; b Department of Dentomaxillofacial Radiology, Faculty of Dentistry, Giresun University, Giresun, Türkiye; c Department of Oral and Maxillofacial Surgery, Faculty of Dentistry, Giresun University, Giresun, Türkiye

**Keywords:** artificial intelligence, dentistry, ethical awareness, data security, anonymization

## Abstract

**Objective:**

This study aimed to evaluate the awareness levels of actively practicing dentists in Türkiye regarding artificial intelligence (AI)-related ethical issues, data security, anonymization, and legal regulations.

**Materials and methods:**

A cross-sectional online survey (Google Forms) used a 12-item questionnaire (4 demographics; 8 awareness domains) rated on a five-point Likert scale. Participants were recruited via snowball sampling. Descriptive statistics, independent-samples *t*-tests, and one-way analysis of variance with Tukey post hoc tests were applied (*p* < 0.05).

**Results:**

A total of 257 dentists participated. Mean domain scores ranged from 2.97 to 3.13; awareness of ethical issues was highest (3.13 ± 1.44) and perception of encryption lowest (2.97 ± 1.45). No significant gender differences were observed. University hospital dentists reported significantly higher awareness of ethical issues and a greater perceived need for anonymization than other institution types (*p* < 0.05). For awareness of Personal Data Protection Law (KVKK), scores were higher in university hospitals and private dental polyclinics than in private practices and public hospitals (*p* < 0.05). Professional experience was associated with differences in perception of encryption, awareness of personal data protection law, awareness of ethical issues, and perceived AI ethical risks (*p* < 0.05); perception of data security and awareness of big data security did not differ significantly (*p* > 0.05).

**Conclusion:**

Dentists demonstrated varying levels of awareness across domains, with higher awareness reported in academic settings. Experience-related patterns differed by domain, indicating the need for focused educational strategies addressing legal and ethical aspects of AI-supported dental practice.

## Introduction

Artificial intelligence (AI)-driven digital technologies are increasingly integrated into contemporary dental practice, particularly in diagnostic imaging, treatment planning, and clinical data management [1–4]. Advances in machine learning and automated image analysis have enabled the processing of large volumes of patient data, enhancing diagnostic efficiency and supporting clinical decision-making processes. As a result, dental professionals are now routinely interacting with AI-supported systems that rely heavily on digital patient records and imaging data [2–4].

Despite these technological benefits, the growing reliance on AI-based systems has introduced significant ethical and legal challenges related to patient data security, privacy protection, and professional accountability [5–7]. The handling of sensitive health data within AI infrastructures raises concerns regarding unauthorized access, secondary data use, insufficient anonymization, and unclear responsibility in the event of data breaches or algorithmic errors [5, 6]. These challenges are particularly critical in healthcare settings, where personal health information is subject to strict legal protections and ethical obligations [7].

International regulatory frameworks, such as the General Data Protection Regulation (GDPR) and the Health Insurance Portability and Accountability Act (HIPAA), establish fundamental principles for the lawful processing, storage, and sharing of personal health data. In Türkiye, the Personal Data Protection Law (KVKK) similarly defines legal responsibilities for healthcare professionals and institutions, emphasizing data minimization, informed consent, and secure data handling practices [8]. However, compliance with these regulations does not depend solely on institutional policies or technical safeguards; it also requires sufficient awareness and understanding among healthcare professionals who directly manage and interact with patient data in daily clinical practice [9].

Existing literature on AI in dentistry has predominantly focused on algorithmic performance, diagnostic accuracy, and technical implementation of AI-based systems [2, 10]. More recently, conceptual discussions addressing ethical principles such as transparency, fairness, and accountability in AI-supported healthcare have emerged [9, 10]. Nevertheless, empirical evidence examining dentists’ self-reported awareness of data security practices, legal regulations, anonymization requirements, and AI-related ethical risks remains limited, particularly in non-academic clinical settings and at the national level [11–13].

Recent publications addressing ethical and legal aspects of AI in dentistry have reported that, although dental professionals generally hold positive attitudes toward AI technologies, considerable uncertainty remains regarding legal responsibilities, data protection regulations, anonymization practices, and accountability in AI-assisted clinical decision-making [11, 12]. Studies conducted in different national contexts have highlighted gaps in awareness related to patient data privacy, anonymization practices, and ethical risk management, particularly outside academic or research oriented clinical environments [12, 13]. These findings suggest that ethical and data security awareness among dental professionals is heterogeneous and context dependent, underscoring the need for country specific empirical investigations [11–15].

Therefore, the present study aims to explore dentists’ self-reported awareness of data security, anonymization, legal obligations, and ethical risks associated with AI-supported clinical practice in Türkiye. By providing an empirical overview of current awareness patterns, this study seeks to inform future educational strategies and policy initiatives aimed at promoting ethically responsible and legally compliant use of AI technologies in dentistry.

## Materials and methods

### Study design and ethical approval

This study was designed as a cross-sectional, descriptive survey aimed at exploring dentists’ self-reported awareness of data security, legal regulations, anonymization practices, and ethical risks associated with AI-supported clinical practice. The study protocol was reviewed and approved by the Afyonkarahisar Health Sciences University Clinical Research Ethics Committee (Decision No: 2025/366). All procedures were conducted in accordance with the principles of the Declaration of Helsinki.

Prior to participation, all respondents were informed about the purpose of the study, the voluntary nature of participation, and data confidentiality. Informed consent was obtained electronically from all participants before they accessed the questionnaire.

### Participants, sampling, and sample size estimation

The study population consisted of dentists actively practicing in Türkiye. Dentists were eligible for inclusion if they were involved in routine clinical practice and regularly used digital patient records and/or radiographic imaging systems.

Participants who did not complete all questionnaire items or who provided duplicate responses were excluded from the analysis. Duplicate entries were identified through technical consistency checks.

An a priori sample size estimation was performed using G*Power software (version 3.1.9.7) to ensure sufficient statistical power for the planned group comparisons. Assuming a medium effect size (Cohen’s d = 0.50 and f = 0.25 for one-way analysis of variance [ANOVA]), an alpha level of 0.05, and a desired statistical power of 0.80, a minimum sample size of approximately 200 participants was required. To account for potential data loss due to incomplete responses or exclusion during data screening, the target sample size was increased by 10%, resulting in a planned inclusion of at least 220 participants.

Following completion of data collection and data screening, a total of 257 valid responses were retained for the final analysis.

### Questionnaire development and content

Data were collected using a structured, self-administered online questionnaire consisting of 12 items (Supplementary Files 1). The first four items assessed demographic characteristics, including age, gender, years of professional experience, and type of institution. The remaining eight items evaluated dentists’ self-reported awareness regarding data security, encryption practices, informed consent, legal regulations related to personal data protection, anonymization requirements, big data security, and ethical risks associated with AI-supported systems.

Each awareness domain was assessed using a single item formulated to represent a distinct conceptual construct. Responses were recorded on a five-point Likert scale ranging from ‘1 = strongly disagree’ to ‘5 = strongly agree’. The questionnaire was designed to assess perceived awareness rather than objectively measured knowledge or technical competence.

### Data collection method

This study was conducted using an online questionnaire consisting of 12 items, developed via Google Forms. In the introduction section of the survey, participants were informed about the aim of the study, data confidentiality principles, and ethical approval. It was clearly stated that participation was entirely voluntary. To proceed with the survey, participants were required to check a confirmation box stating, ‘I voluntarily agree to participate in this study and consent to the anonymous use of my survey data’.

The data collection process employed a snowball sampling method. The survey link was distributed to dentists via email, social media platforms, and various online communication channels. Participants were also encouraged to share the link with other dentists in their networks to increase participation.

### Statistical analysis

The collected data were analyzed using IBM SPSS Statistics software (version 27.0; IBM Corp., Armonk, NY, USA). The normality of numerical data distribution was assessed using the Shapiro–Wilk test. Descriptive statistics were calculated and reported as frequency (*n*), percentage (%), mean (X^¯^), and standard deviation (SD).

To examine the relationships between participants’ demographic characteristics (gender, years of professional experience, and type of institution) and their levels of ethical awareness, perceptions of data security, and familiarity with legal regulations, independent samples *t*-tests were used for comparisons between two groups, and one-way ANOVA was used for comparisons involving more than two groups. When statistically significant differences were detected in ANOVA, post hoc pairwise comparisons were performed using the Tukey test.

A two-sided *p*-value of < 0.05 was considered statistically significant for all analyses.

## Results

A total of 257 dentists participated in the online survey (female: *n* = 149, 58%; male: *n* = 108, 42%). The mean age of the participants was 35.92 ± 9.83 years. The largest proportion of participants had 11–20 years of professional experience (31.5%), whereas dentists with ≥ 21 years of experience constituted the smallest group (17.2%). Regarding institutional affiliation, most participants were employed in private dental polyclinics (36.2%), followed by private practices (22.9%), public hospitals (22.2%), and university hospitals (18.7%) (Table 1).

**Table 1:** Demographic characteristics of the study participants.

Variables		*n*	%
Gender	Female	149	58
	Male	108	42
Years of professional experience	0–5 years	60	23.3
	6–10 years	72	28
	11–20 years	81	31.5
	≥ 21 years	44	17.2
Type of institution	Private practice	59	22.9
	Private dental polyclinic	93	36.2
	Public hospital	57	22.2
	University hospital	48	18.7
Total		257	100

Data are presented as number (*n*) and percentage (%).

Across the eight assessed domains, mean awareness scores were distributed within the mid-to-upper range of the five-point Likert scale, ranging from 2.97 to 3.13. The highest mean score was observed for awareness of ethical issues (3.13 ± 1.44), whereas the lowest score was recorded for perception of encryption (2.97 ± 1.45) (Table 2).

**Table 2:** Mean awareness scores across individual domains.

Domains	Mean ± SD
Perception of Data Security	3.02 ± 1.41
Perception of Encryption	2.97 ± 1.45
Awareness of Informed Consent	3.02 ± 1.38
Awareness of Personal Data Protection Law	2.98 ± 1.45
Awareness of Ethical Issues	3.13 ± 1.44
Perception of AI Ethical Risks	3.08 ± 1.41
Perceived Need for Anonymization	2.98 ± 1.39
Awareness of Big Data Security	3.11 ± 1.41

Data are presented as mean ± standard deviation (SD).

Comparisons by gender revealed no statistically significant differences across any of the assessed domains (all *p* > 0.05; Table 3). Female dentists had slightly higher mean scores for perception of data security (3.05 ± 1.40 vs. 2.93 ± 1.44) and perception of encryption (3.12 ± 1.42 vs. 2.76 ± 1.53), whereas male dentists had slightly higher mean scores for awareness of big data security (3.27 ± 1.38 vs. 2.99 ± 1.42) and awareness of informed consent (3.09 ± 1.39 vs. 2.97 ± 1.34); however, none of these differences reached statistical significance (*p* = 0.456, *p* = 0.084, *p* = 0.086, and *p* = 0.480, respectively). Scores for awareness of personal data protection law, awareness of ethical issues, perception of AI related ethical risks, and perceived need for anonymization were similar between genders (*p* = 0.452, *p* = 0.226, *p* = 0.947, and *p* = 0.123, respectively) (Table 3).

**Table 3:** Awareness domain scores by gender.

Domains	Male	Female	*P* *
	Mean ± SD	Mean ± SD	
Perception of Data Security	2.93 ± 1.44	3.05 ± 1.40	0.456
Perception of Encryption	2.76 ± 1.53	3.12 ± 1.42	0.084
Awareness of Informed Consent	3.09 ± 1.39	2.97 ± 1.34	0.480
Awareness of Personal Data Protection Law	2.90 ± 1.47	3.03 ± 1.43	0.452
Awareness of Ethical Issues	3.02 ± 1.45	3.21 ± 1.39	0.226
Perception of AI Ethical Risks	3.07 ± 1.40	3.09 ± 1.41	0.947
Perceived Need for Anonymization	2.81 ± 1.35	3.09 ± 1.40	0.123
Awareness of Big Data Security	3.27 ± 1.38	2.99 ± 1.42	0.086

* Independent samples *t*-test.

When analyzed according to type of institution, statistically significant differences were identified in three of the eight domains. Awareness of personal data protection law (KVKK) differed across institutions (*p* = 0.016), with higher scores in university hospitals (3.60 ± 1.20) and private dental polyclinics (3.40 ± 1.30) than in private practices (2.70 ± 1.40) and public hospitals (2.90 ± 1.50). Awareness of ethical issues was significantly higher in the university hospital group than in all other institution types (*p* = 0.041). Perceived need for anonymization was also significantly higher among university hospital dentists than among all other institution types (*p* = 0.023). No statistically significant differences were observed across institution types in the remaining domains (all *p* > 0.05) (Table 4).

**Table 4:** Awareness domain scores by type of institution.

Domains	Private practice	Private dental polyclinic	University hospital	Public hospital	*P* *
	Mean ± SD	Mean ± SD	Mean ± SD	Mean ± SD	
Perception of Data Security	2.95 ± 1.39	2.91 ± 1.43	3.12 ± 1.45	3.06 ± 1.37	0.819
Perception of Encryption	3.00 ± 1.49	2.88 ± 1.55	3.07 ± 1.40	2.98 ± 1.33	0.890
Awareness of Informed Consent	3.16 ± 1.36	3.02 ± 1.37	2.85 ± 1.44	3.06 ± 1.41	0.679
Awareness of Personal Data Protection Law	2.70 ± 1.40^b^	3.40 ± 1.30^a^	3.60 ± 1.20^a^	2.90 ± 1.50^b^	0.016
Awareness of Ethical Issues	2.85 ± 1.35^c^	3.10 ± 1.45^b^	3.65 ± 1.25^a^	3.20 ± 1.40^b^	0.041
Perception of AI Ethical Risks	3.28 ± 1.44	3.15 ± 1.35	2.76 ± 1.47	3.11 ± 1.42	0.225
Perceived Need for Anonymization	3.00 ± 1.40^b^	3.20 ± 1.35^b^	3.80 ± 1.10^a^	3.10 ± 1.50^b^	0.023
Awareness of Big Data Security	3.19 ± 1.30	3.21 ± 1.47	3.03 ± 1.38	2.98 ± 1.45	0.596

* One-way ANOVA with Tukey post hoc test; different superscript letters indicate statistically significant differences between groups (p < 0.05).

Analyses based on years of professional experience revealed statistically significant differences in four domains. Perception of encryption was highest among dentists with 11–20 years of experience (3.72 ± 1.41), differing significantly from the 0–5 year (2.92 ± 1.46) and 6–10 year (3.25 ± 1.46) groups (*p* = 0.029). Awareness of personal data protection law varied significantly across experience groups (*p* = 0.037), with the lowest mean score observed among dentists with ≥ 21 years of experience (2.64 ± 1.31). A non-linear pattern was observed for awareness of ethical issues, with higher mean scores in the 0–5 year (3.33 ± 1.35) and ≥ 21 year (3.16 ± 1.51) groups and the lowest score in the 11–20 year group (2.98 ± 1.53) (*p* = 0.008). In addition, perception of AI-related ethical risks showed a significant decline with increasing professional experience, from 3.37 ± 1.37 in the 0–5 year group to 2.48 ± 1.41 among dentists with ≥ 21 years of experience (*p* = 0.007). No statistically significant differences were found across experience groups for perception of data security, awareness of informed consent, perceived need for anonymization, or awareness of big data security (all *p* > 0.05) (Table 5).

**Table 5:** Awareness domain scores by years of professional experience.

Domains	0–5 Years	6–10 Years	11–20 Years	≥ 21 Years	*P* *
	Mean ± SD	Mean ± SD	Mean ± SD	Mean ± SD	
Perception of Data Security	3.11 ± 1.41	3.15 ± 1.45	2.85 ± 1.32	2.86 ± 1.5	0.307
Perception of Encryption	2.92 ± 1.46^c^	3.25 ± 1.46^b^	3.72 ± 1.41^a^	3.07 ± 1.48^bc^	0.029
Awareness of Informed Consent	3.10 ± 1.43	3.14 ± 1.36	2.88 ± 1.37	2.98 ± 1.41	0.717
Awareness of Personal Data Protection Law	3.21 ± 1.48^a^	3.14 ± 1.49^a^	3.07 ± 1.46^a^	2.64 ± 1.31^b^	0.037
Awareness of Ethical Issues	3.33 ± 1.35^a^	3.12 ± 1.38^ab^	2.98 ± 1.53^b^	3.16 ± 1.51^ab^	0.008
Perception of AI Ethical Risks	3.37 ± 1.37^a^	3.14 ± 1.31^a^	2.68 ± 1.52^b^	2.48 ± 1.41^b^	0.007
Perceived Need for Anonymization	2.75 ± 1.36	3.19 ± 1.39	2.99 ± 1.36	2.91 ± 1.48	0.684
Awareness of Big Data Security	3.05 ± 1.55	2.97 ± 1.32	3.28 ± 1.41	3.07 ± 1.35	0.629

* One-way ANOVA with Tukey post hoc test; different superscript letters indicate statistically significant differences between groups (p < 0.05).

## Discussion

This study aimed to evaluate dentists’ self-reported awareness of data security and ethical issues related to AI-supported digital health applications. Specifically, awareness levels regarding personal data protection, encryption, big data security, anonymization, and AI-related ethical risks were examined, together with the potential influence of demographic variables. The findings demonstrated that general ethical awareness was relatively higher than awareness of data security components.

Ethics, as a philosophical and professional framework, constitutes a foundational pillar of dental practice, encompassing moral responsibility, professionalism, and social accountability [16]. The integration of AI into clinical workflows inevitably introduces novel ethical challenges that extend beyond technical performance, involving responsibility allocation, data governance, and patient autonomy [9, 12]. In the present study, the highest mean scores were observed in ethical awareness, big data security, and perception of AI-related ethical risks. This pattern suggests that dentists may more readily recognize abstract ethical principles than specific data security. Similar observations have been reported by Ducret and Mörch, who emphasized that healthcare professionals often acknowledge concepts such as transparency, fairness, and accountability, while exhibiting uncertainty regarding the operationalization of these principles in daily practice [9]. Likewise, Kim et al. highlighted that although ethical principles are commonly addressed in dental curricula, structured education focusing on AI-specific ethical dilemmas remains limited [13]. Consequently, heightened ethical concern may reflect uncertainty rather than comprehensive ethical competence. Vinayahalingam and Thiem further noted that accountability in clinical errors and data privacy represent the most prominent ethical concerns among dental professionals confronted with AI-based systems [17]. The lack of standardized ethical frameworks and formal AI training has also been shown to intensify ambiguity and apprehension regarding responsible AI use in dentistry [18].

In line with these findings, Engelschalk et al. demonstrated that dental professionals frequently perceive ethical and regulatory aspects of AI as insufficiently defined, particularly regarding responsibility allocation, data ownership, and compliance with legal frameworks, reinforcing the notion that ethical concern often arises from structural uncertainty rather than informed confidence [19].

Gender-based comparisons revealed no statistically significant differences across the examined domains; however, female participants consistently demonstrated higher mean scores in several dimensions, including perception of encryption, ethical awareness, and perceived need for anonymization. Although these differences did not reach statistical significance, the observed trend suggests a potential gender-related sensitivity toward ethical and data protection issues. Previous survey-based research has similarly reported that female dental professionals tend to express stronger ethical concerns and greater expectations for transparency and accountability in AI-assisted decision-making processes [11]. Moreover, broader studies on attitudes toward AI across healthcare and educational settings have indicated that gender may influence ethical risk perception and privacy-related concerns [20]. While definitive conclusions cannot be drawn from the present data, gender may represent a relevant factor shaping ethical sensitivity, warranting further investigation in larger and more diverse samples.

Supportive evidence from dental education contexts indicates that female participants may exhibit greater concern regarding ethical and regulatory implications of AI, particularly in relation to patient data protection and informed consent, suggesting that gender-related differences in ethical sensitivity may emerge even before professional practice [21].

Institutional affiliation emerged as a significant determinant of awareness levels. Dentists working in university hospitals demonstrated significantly higher ethical awareness and a stronger perceived need for anonymization than other institution types; additionally, awareness of KVKK was higher in university hospitals and private dental polyclinics than in private practices and public hospitals. These findings suggest that institutional culture, educational exposure, and engagement with research-oriented environments may enhance ethical and legal awareness. Previous studies have reported that academic settings provide greater exposure to digital health systems, regulatory discussions, and ethical deliberation, thereby fostering more informed attitudes toward data governance and AI-related risks [14, 15]. In contrast, the comparatively lower awareness observed in private hospital and clinic settings highlights potential gaps in continuing education and institutional policy frameworks addressing digital ethics and data security.

Recent empirical data further support this interpretation. Al-Khalifa et al. found that participants affiliated with academic institutions exhibited higher levels of awareness regarding ethical governance, regulatory compliance, and responsible AI use, emphasizing the role of structured educational environments in shaping ethical literacy related to AI technologies [22].

Professional experience was inversely associated with several awareness dimensions. Dentists with fewer years of experience exhibited higher levels of ethical awareness, knowledge of data protection regulations, and perception of AI-related ethical risks compared with more senior practitioners. This pattern may reflect generational differences in exposure to digital technologies and formal education on AI-related topics. Recent studies have indicated that younger healthcare professionals and students encounter AI concepts earlier during training and may therefore develop greater digital literacy and ethical awareness [20]. Conversely, senior professionals may adopt a more cautious stance toward emerging technologies, relying primarily on clinical experience rather than technical infrastructure knowledge [23]. These findings align with the broader literature emphasizing that successful integration of AI into dental practice depends not only on technological advancement but also on ethical responsibility, transparency, trust, and robust data protection mechanisms [10].

This study has several limitations. First, the use of a self-reported questionnaire may have introduced social desirability bias, which could have led participants to overestimate their levels of awareness. In addition, as the study was conducted within a single national context, the generalizability of the findings to healthcare systems with different cultural and legal frameworks may be limited.

## Conclusion

This study examined dentists’ levels of awareness in Türkiye regarding data security, anonymization, and ethical issues related to AI. While no significant differences were observed according to gender, institutional setting and professional experience were found to be associated with awareness levels. Dentists working in university hospitals reported higher awareness of personal data protection legislation (KVKK), ethical issues, and anonymization, whereas experience-related patterns varied across certain domains. These findings indicate a need for structured professional education focusing on legal regulations, anonymization, and basic data security, particularly for dentists working outside academic settings.

## Disclosure statement

No potential conflict of interest was reported by the authors.

## Data availability

The data that support the findings of this study are available on request from the corresponding author.

## Ethics approval

The study received ethical approval from the Afyonkarahisar Health Sciences University Clinical Research Ethics Committee, with a decision numbered 2025/366.

## Supplementary Material


