# Supplementary material for: Dentists’ awareness of data security and ethical issues during the transition to artificial intelligence-driven clinical practice
Source: Acta Odontol Scand. 2026 Feb 4;85:45419. doi: 10.2340/aos.v85.45419 (PMC12884369; doi:10.2340/aos.v85.45419)
Supplement: Supplementary file 1 [file AOS-85-45419-s1.pdf]

Supplementary material has been published as submitted. It has not been copyedited or typeset by Acta Odontologica Scandinavica.

Survey: Awareness of Artificial Intelligence, Ethical Issues and Data Security Among Dentists

Section 1: Demographic Information

1. Age: __________

2. Gender:

☐ Female
☐ Male

3. How many years of professional experience do you have in the field of dentistry?

☐ 0–5 years
☐ 6–10 years
☐ 11–20 years
☐ More than 21 years

4. What type of institution do you currently work in?

☐ Private Practice
☐ Private Dental Polyclinic
☐ Public Hospital
☐ University Hospital

Section 2: Awareness of Data Security and Ethical Issues Related to Artificial Intelligence

5. Patient data is securely stored at the institution/clinic where I work.

☐ Strongly agree
☐ Agree
☐ Neutral
☐ Disagree
☐ Strongly disagree

6. Secure data transfer and storage practices (e.g., encryption and/or two-factor authentication) are implemented at the institution/clinic where I work.

☐ Strongly agree
☐ Agree
☐ Neutral

☐ Disagree
☐ Strongly disagree

7. Informed consent is obtained from patients before their data are shared with third parties (e.g., external companies or platforms).

☐ Strongly agree
☐ Agree
☐ Neutral
☐ Disagree
☐ Strongly disagree

8. I am knowledgeable about the legal regulations regarding patient data protection in Türkiye (e.g., Personal Data Protection Law (KVKK), Patient Rights Regulation).

☐ Strongly agree
☐ Agree
☐ Neutral
☐ Disagree
☐ Strongly disagree

9. I am knowledgeable about potential ethical issues related to AI supported applications (e.g., privacy breaches, bias, and lack of transparency).

☐ Strongly agree
☐ Agree
☐ Neutral
☐ Disagree
☐ Strongly disagree

10. The transfer of patient data to third parties for AI applications poses ethical risks.

☐ Strongly agree
☐ Agree
☐ Neutral
☐ Disagree
☐ Strongly disagree

11. Patient data used in artificial intelligence systems must be anonymized.

☐ Strongly agree
☐ Agree

☐ Neutral
☐ Disagree
☐ Strongly disagree

12. The use of large patient datasets to train AI models may pose additional risks to data security.

☐ Strongly agree
☐ Agree
☐ Neutral
☐ Disagree
☐ Strongly disagree
